# Transplant of the Abdominal Rectus Fascia in rats, first report of a novel experimental technique

**DOI:** 10.1016/j.intf.2024.100019

**Published:** 2024-10-10

**Authors:** Jeremias E. Moreira, Pablo Stringa, Marco Santillán Pazmiño, Anastasios Giannou, Constanza Arriola Benitez, Martín Rumbo, María Virginia Gentilini, Gabriel E. Gondolesi

**Affiliations:** a Instituto de Medicina Traslacional, Trasplante y Bioingeniería (IMETTyB), Universidad Favaloro-CONICET, Ciudad Autónoma de Buenos Aires, Solis 453, Ciudad Autónoma de Buenos Aires, Argentina; b Instituto de Estudios Inmunológicos y Fisiopatológicos (IIFP) - CONICET - Facultad de Ciencias Exactas, La Plata, Provincia de Buenos Aires, Blvd. 120 1489, La Plata, Provincia de Buenos Aires, Argentina; c Cátedra de Trasplante, Facultad de Ciencias Médicas, Universidad Nacional de La Plata, Solis 453, Ciudad Autónoma de Buenos Aires, Argentina; d Hamburg Center for Translational Immunology (HCTI), University Medical Center Hamburg-Eppendorf, 20246 Hamburg, Germany; e Servicio de Cirugía General, Trasplante Hepático, Pancreático e Intestinal. Hospital Universitario Fundación Favaloro, Ciudad Autónoma de Buenos Aires, Av. Belgrano 1746, Ciudad Autónoma de Buenos Aires, Argentina

**Keywords:** ARF, Non-vascularized, Tissue, Transplant and intestine

## Abstract

**Background:**

After intestinal or multivisceral transplant, closing the abdominal wall can be challenging, as negative pressure dressing or synthetic meshes pose risks like infections and fistulas. Clinical practice has evolved from vascularized abdominal wall transplants to non-vascularized Abdominal Rectus Fascia (TxARF). Although it was successful, many immunological aspects remain unknown, highlighting the need for further research.

**Methods:**

The technique was developed by reproducing the technical aspects of the procedure described for humans in rats (Wistar and Sprague Dawley). Twenty-six Isogenic and Allogeneic TxARF procedures were performed and followed until 30 and 120 post-transplant days (PTD). The non-implanted fascias served as a control group. Rats were then re-assessed for engraftment on 7, 11, 30 and 120 PTD. Fascia samples were taken to assess neovascularization by quantifying cell composition and blood vessels using H&E and Orcein staining.

**Results:**

All animals (N=26) survived at 30 and 120 PTD, with 4 (15.4 %) developed subcutaneous serum collection. Upon reoperation, grafts showed neovascularization. No adhesions were observed between the intestines and the grafts. The principal cell compound of the fascia was represented by Fibroblasts (18.35 cells/field) and Myocytes (6.57 cells/field). A significant increment of the number of blood vessels were observed during the period studied (p=0.046).

**Conclusions:**

Our report on TxARF in rats, proves the feasibility of this experimental and translational model, showing similar results to those published in the clinical field. Further studies are required to evaluate the immunogenicity as well as the changes in ARF overtime.

## Introduction

One of the major challenges of open surgery has been the successful closure of the abdominal wall. Its failure leads to acute or chronic complications that will increase patients' perioperative morbidity and impair quality of life, independently of the original procedure [1]. In patients requiring an abdominal organ transplant, the risk is increased not only by the severity of a long-term disease but also by the type of incision required, which may result in multiple scars or ostomies from previous surgical procedures [2], [3], [4].

When candidates for isolated intestine, combined liver-intestine, or a multivisceral transplant are referred for transplant evaluation, the closure of the wall should be considered a crucial aspect of the process, especially in patients with short bowel syndrome who have a significant loss of the abdominal domain. After evaluation, and pending on each individual country regulation, the Abdominal Rectus Fascia (ARF) needs to be requested in addition to the organs required to be transplanted [5], [6], [7], [8].

Over time, different strategies have been proposed to overcome the discrepancy between the new visceral content and the lack of abdominal domain [9], [10], such as the use of a pediatric or “a pediatric size” adult donors, graft size reduction, the use of a vascularized rotational flap from the tight, the use of absorbable synthetic meshes, acellular dermal matrices, or negative pressure dressings as part of the wound care. All have different types of complications reported, and abdominal wall transplantation appeared to be the most advanced. Therefore, it was seen as the new “gold standard” [3], until the transplant of the non-vascularized fascia of the Abdominal Rectus muscle was described (TxARF) [11], [12].

The benefits of using the ARF, include: a) higher recipient acceptability from an aesthetic point of view, b) reduced morbidity and mortality rates, c) no need for rejection monitoring, and d) the possibility of using fascia from a second donor to replace the previous one, among others [2], [13]. Two possible procedures were described: the so-called “Favaloro’s technique”, which aimed at implanting only the anterior fascia [11]; and the “Miami’s technique”, which uses both the anterior and posterior fascia. The latter provides greater strength by maintaining the peritoneal layer minimizing the risk of developing adhesions [14], [15]. Currently, the TxARF has been used in adults and pediatric recipients of intestinal containing grafts [16], as well as in recipients of other solid organs such as the liver or kidney who develop large ventral defects. Despite the current use of this technique, many immunological and biological aspects remain unknown, highlighting the necessity for further basic and translational science research [17]. Therefore, we decided to develop an experimental rat model for TxARF as a novel microsurgical procedure aiming to improve its knowledge and understanding.

## Materials and methods

**Fig. 1:**
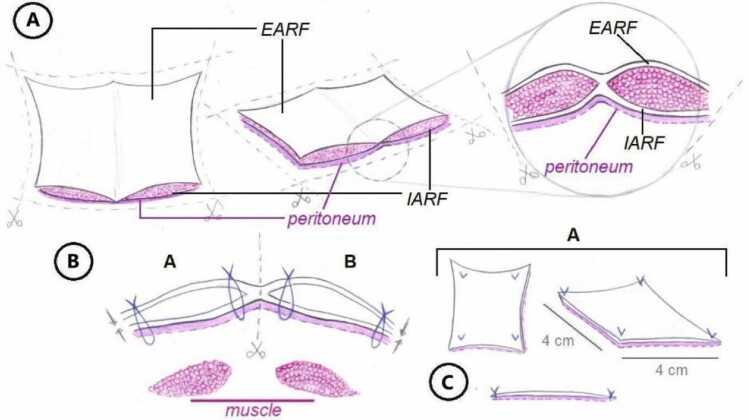
A and B- Dissection and fixation of the External Abdominal Rectus Fascia (EARF) and the Internal Abdominal Rectus Fascia (IARF) with Peritoneum. C- Final state of the tissue to implant in the recipient rat.

The procedures were approved by the internal review board and ethics committee for the care and use of laboratory animals (CICUAL UF 2021-016 PICT 2016-3677, Ext. CICUAL UF 2019-004) and the ARRIVE 2.0 guidelines for animal studies. The technical aspects were carried out by reproducing the “Miami technique” used in humans (Fig. 1). Sixteen TxARF procedures were performed and followed up until 30 PTD (8 isogenic and 8 allogeneic TxARF), 10 TxARF procedures were followed up until 120 PTD (5 isogenic and 5 allogeneic TxARF). The donor rats (Sprague Dawley or Wistar) had an average weight of 385 ± 30 g, and the recipients, both Sprague Dawley and Wistar, had an average weight of 320 ± 38 g. Male and female rats were used. Un-implanted ARF served as a control group (N = 6). Transplanted rats were kept on a reverse light cycle with 12-h light/dark, 24 °C temperature, food and water *ad libitum*. All procedures were performed under sterile conditions, and all rats were induced and maintained under mask inhalation anesthesia with Isoflurane (5 % and 2 % respectively) and oxygen flow of 2–3 l/h. Tramadol 40 mg/kg was applied subcutaneously as pretreatment. The recipients received local subcutaneous lidocaine (2 %) 0.5 ml total, Ceftriaxone 70 mg/kg subcutaneously, Saline SC 3 to 5 ml/kg subcutaneously in two stages throughout the surgery and the use of eye ointment.

### Donor operation

**Fig. 2:**
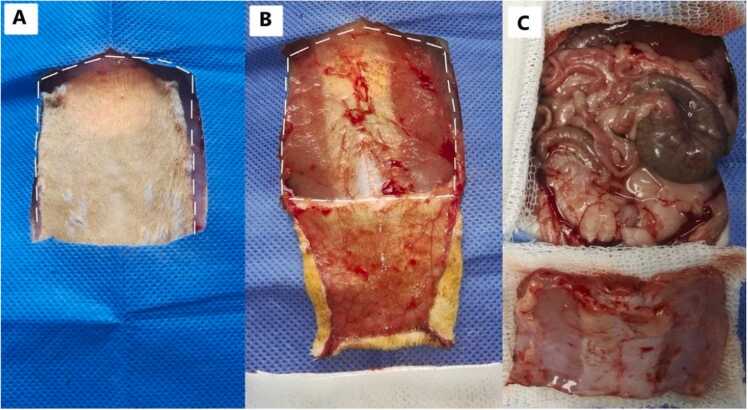
A- The white lines induce the cut directions of the skin in the donor rat. B- The white lines induce the cut directions of the abdominal wall in the donor rat. C- Abdominal muscle wall ready for dissection.

The donor rat was positioned in supine position. Two subcostal skin incisions and two lateral paramedian incisions (right and left) were made from the hypochondrium to the ipsilateral inguinal area. After making the incisions, skin was separated from the ARF and retracted (Fig. 2). The displaced skin was covered with sterile gauze, and similar incisions were made following the lateral edge of the rectum muscles, extending up to the level of the peritoneum (Fig. 2). Finally, a horizontal incision was made proximal to the pubis.

### Back-table surgery

**Fig. 3:**
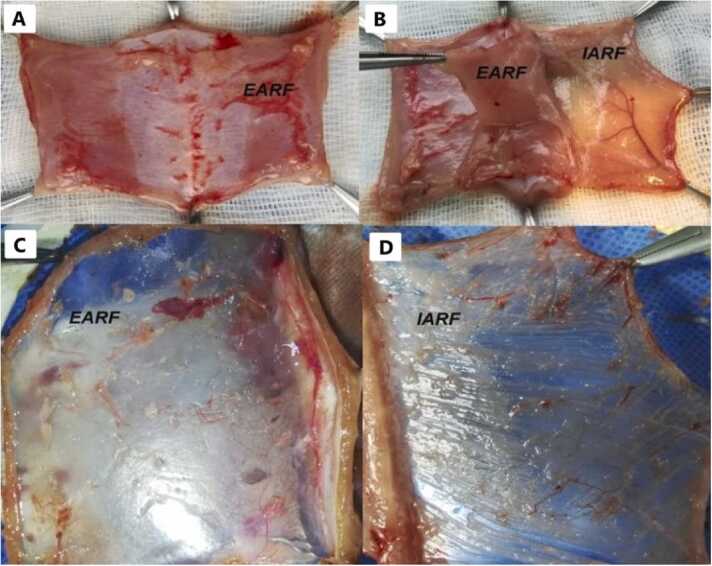
A and B- Appear of Abdominal muscle wall without dissection. C and D- External Abdominal Rectus Fascia (EARF) and Internal Abdominal Rectus Fascia (IARF) with Peritoneum.

The extracted tissue was placed on sterile gauze, and then 6 Halsted haemostatic forceps were used to fix each quadrant of the tissue (Fig. 3), Initially, the anterior and posterior layers of the graft were separated at the external border to completely remove the Abdominal Rectus muscle, using a microscope at 4 × magnification, with utmost care to avoid fascia damage (Fig. 3). Once dissection was completed, the anterior and posterior fascias were joined by placing a 7-0 Prolene® sutures. Finally, incisions were made in the fascia to obtain a graft size of approximately 4 cm × 4 cm extended. Each graft was then placed in the Histidine-Tryptophan-Ketoglutarate Solution (HTK) preservation in a sterile Petri dish at 4 °C, for 30 min, before engraftment.

### Recipient procedure

**Fig. 4:**
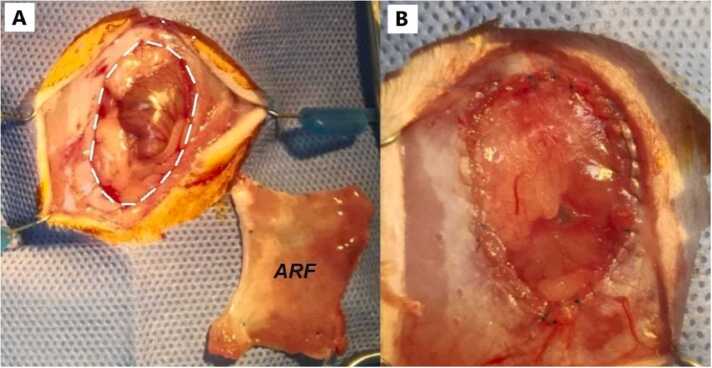
A- Extraction of a portion of the native abdominal wall (white line), (External Abdominal Fascia, Internal Abdominal Fascia and Peritoneum) ready to implant. B- Graft implantation in the recipient ARF.

The recipient rat was placed in dorsal decubitus, and a midline xypho-pubic incision was made. The Abdominal Rectus muscles were removed, including the peritoneal layer, to create a space in the abdominal wall for implantation. Once haemostasis was achieved, the graft was brought into the surgical field, adjusted accordingly, and grafted using two 7-0 Prolene® running sutures encompassing both native fasciae (Fig. 4). Finally, the skin was closed with a single strain suture of 3-0 Prolene ®.

### Post-transplant course

Once transplantation was completed, recipients were individually housed for 12 days, and then classified into different groups for follow up at 30 and 120 PTD. During the first 3–4 days after surgery, feeding consisted of 1/4 an apple without seeds and balanced rat food. Thereafter, balanced rat food was provided for up to 30 and 120 PTD.

**Fig. 5:**
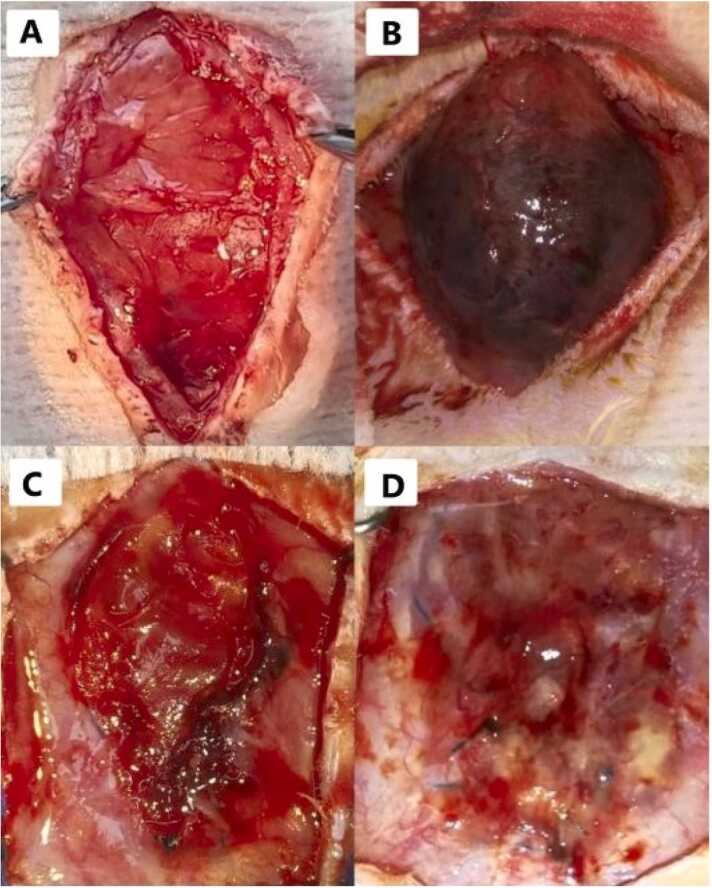
A- Appearance of the isogenic TxARF at 7 PTD. B- Appearance of the isogenic TxARF at 11 PTD (with presence of seroma). C- Appearance of the allogeneic TxARF on 7 PTD. D- Appearance of the allogeneic TxARF on 11 PTD.

**Fig. 6:**
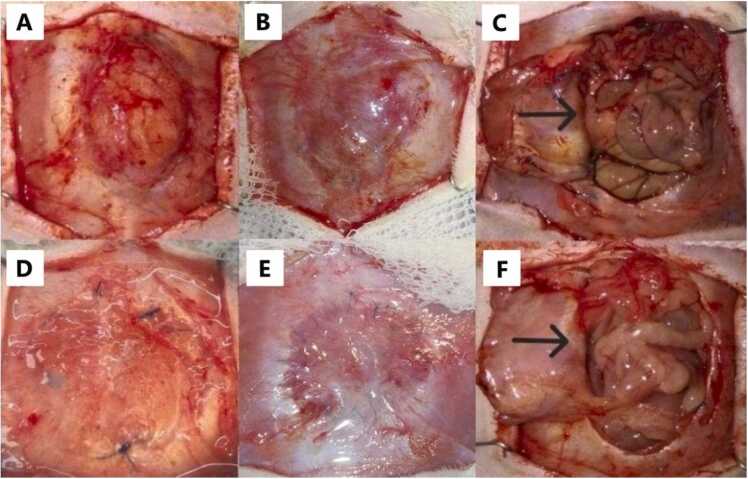
A- Isogenic TxARF on day 30. B- Isogenic TxARF at 120 PTD. C- Macroscopic appearance of the fascia and abdominal visceras in isogenic TxARF. D- Allogeneic TxARF at 30 PTD. E- Allogeneic TxARF at 120 PTD. F- Macroscopic appearance of the fascia and abdominal visceras in allogeneic TxARF. The arrows indicate the non-adhesions between the graft and the intestine.

All recipients were monitored under veterinary inspection and control. Weight was controlled, and Tramadol 40 mg/kg and Ceftriaxone 70 mg/kg were applied subcutaneously every 24 h for 7 PTD. Observations and photographs of the grafts were recorded on days 7, 11 (Fig. 5), 30 and 120 PTD (Fig. 6) for each strain, focusing solely on the skin. Recipient rats were sacrificed on 30 or 120 PTD, and fascia samples were collected. Samples of the Control ARF underwent analysis with H&E staining. Ten fields were examined at 40x magnification, and cells were identified and quantified for each control sample. Orcein staining was performed to assess the presence of blood vessels within the elastic fibers of all TxARF samples. Each histological sample stained with Orcein was divided into quadrants, and 5 random quadrants were selected for each fascia. The number of blood vessels present at 40x magnification in each quadrant was counted, and the median with its range was calculated for each sample and group with a 95 % confidence interval. Statistical analysis was conducted using Kruskal-Wallis and normality tests.

## Results

**Fig. 7:**
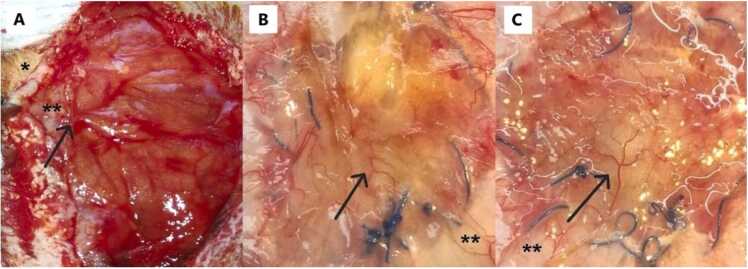
A- Appearance of the graft at 7 PTD (Isogenic TxARF). B- Appearance of the graft at 11 PTD (Allogeneic TxARF). C- Appearance of the graft at 30 PTD (Allogeneic TxARF). Skin (*). Native Abdominal muscle wall (**). The arrows indicate neo-vascularization in the graft.

The donor and back-table for the ARF required 5.5 ± 0.5 h, while the engraftment 35 ± 10 min. None of the recipients suffered evisceration, or ventral defects. During the course of visual observation of the grafts, neither signs of ischemic changes, nor an increase in tissue stiffness were observed, but progressive development of neo-vascularization from the recipient to the donor tissue (Fig. 7). Four recipients (15.4 %) developed subcutaneous serum collections, diagnosed at day 5 and 11, all of them were the isogenic TxARF and successfully drained.

**Fig. 8:**
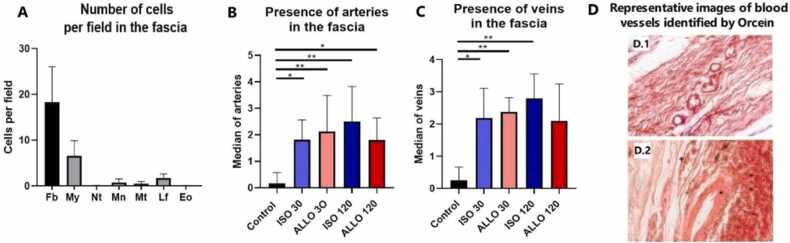
A- Number of cells per 40x field in the control fascia: Fb (Fibroblast), My (Myocytes), Nt (Neutrophils), Mn (Monocytes), Mt (Mast cells), Lf (Lymphocytes), Eo (Eosinophils); B- Presence of arteries in the fascia: * (p ≤ 0.0466), ** (p ≤ 0.0091); C- Presence of veins in the fascia: * (p = 0.0191), ** (p ≤ 0.0061); D- Representative images of blood vessels identified by Orcein: D.1: Small blood vessels, D.2: Big blood vessels, **+** (Venous wall), * (Arterial wall).

Survival at 30 and 120 PTD was accomplished by all the recipients (100 %). The control fascias presented an average of 18.35 ± 7.68 (64.29 %) Fibroblasts, 6.57 ± 3.34 (21.25 %) Myocytes, 1.75 ± 0.88 (5.34 %) Lymphocytes, 0.75 ± 0.78 (1.17 %) Monocytes, 0.45 ± 0.52 (1.17 %) Mast cells, 0.07 ± 0.08 (0.19 %) Neutrophils and 0.03 ± 0.08 (0.08 %) Eosinophils per field at 40x (Fig. 8.A). At 30 PTD, the number of arteries identified by Orcein in the fascia graft was significantly higher in isogenic TxARF (Median 2, Range 2; p ≤ 0.0466) and in allogeneic TxARF (Median 2, Range 4; p ≤ 0.0091) in comparison with the control group (Median 0, Range 1). Similar differences with the control group was observed in isogenic TxARF (Median 2, Range 3.5; p ≤ 0.009) and allogeneic TxARF (Median 2, Range 2; p ≤ 0.0466) at 120 PTD (Fig. 8.B). On the other hand, at 30 PTD the number of veins was significantly greater in both isogenic TxARF (Median 2, Range 2.5; p = 0.0191) and allogeneic TxARF (Median 2.25, Range 1; p ≤ 0.0061) compared to the control group (Median 0, Range 1). However, at 120 PTD the isogenic TxARF group presented significant differences (Median 3, Range 1.5; p ≤ 0.0061) while the allogeneic TxARF group did not reach statistical significance (Median 2, Range 2.5; p = 0.06) (Fig. 8.C).

## Discussion

After the description of the TxARF, its use has been widely expanded. In one of the latest reviews [2], it was shown that the reported number of cases exceeded the number of vascularized abdominal wall transplants worldwide. The current numbers will soon be reported as the result of a survey available on the IIRTA web page (www.tts.org/irta-about/). The original technique has also evolved to be used not only in intestinal transplant recipients but also in recipients of other organs such as liver [19] or kidney transplants who develop ventral hernias, or as a vascularized composite graft with the liver for use in patients with the Prune-Belly syndrome [18]. However, despite recent advances, there are still several unanswered questions regarding potential applications of the technique in non-transplant patients. Among these, the risk of inducing de-novo donor specific antibodies through the procedure has not been described, because the fascia is mostly used simultaneously with an isolated intestinal or a MTV graft. For these reasons, we decided to develop a microsurgical translational model from the clinical field to the basic science. We have identified and described pitfalls in the three main stages of the procedure: procurement, bench preparation, and engraftment.

The ARF should be immediately placed in the Petri dish with a preservation solution, (HTK®, was used in our experiments) at 4 °C. During the bench/back-table period, both fasciae should be dissected, and the Abdominal Rectus muscles removed. The back-table starts with separating the external and internal fascia, followed by complete removal of the muscle fibers using a microscope and left-hand forceps. It is important to highlight that the areas with aponeurosis of the abdominal rectus muscle will represent the greatest challenge due to the strong muscular attachment to the fascia. The greatest risk of ARF rupture occurs during the dissection of the lateral portions of the external fascia, potentially affecting its use. For this reason, the edges should be trimmed from the tissue intended for implantation toward the end of the dissection. At this stage, it is essential to keep the abdominal wall throughout the process, using sterile saline solution to prevent the hardening and rupture of the fascia. To optimize the resources, the ARF can be divided by transecting it along the “linea alba”, allowing for 2 transplants to be performed and thereby reducing the number of donor animals. Implementing this variation in two of the recipients significantly reduced the number of required animals and time. A cold light source is recommended to avoid excessive desiccation.

This preliminary report is the proof of concept to demonstrate its feasibility, with the aim of expanding it to other microsurgical laboratories to address various aspects that require further investigation.

For the engraftment in the recipient, we found it important to extract of most central and ventral area of the abdominal wall the Abdominal Rectus muscle, with the complete external and internal fascia and the peritoneum, as well as a small portion of the transverse and oblique muscles. This approach avoids the need for incisions that might require transection and suturing of large blood vessels, such as the cranial and caudal epigastric arteries, to prevent future risks such as hemorrhage or ischemic events that could impact the recipient's survival. It is recommended to perform these resections using scissors. The extensive dissection required after the skin incision, necessary for exposing the entire abdominal wall, is responsible for the development of post-transplant seromas, which have been the most relevant complication observed in this initial experience. The use of electrocautery could replace scissors and reduce the presence of small hemorrhages. However, the thermal effect on the small muscles of the abdominal wall of the recipient rat could decrease the vitality of the cauterized edges where the implant will be performed. This might facilitate dehiscence processes between the implanted fascia and the native abdominal wall, leading to the potential development of ventral hernias or post-surgical. The bench of the fascia remains longer than the engraftment (mean: 35 min). Post-surgical recovery follows protocols like those used for other types of transplants in rodents. The use of a non-continuous suture for the skin was essential to prevent the recipients from removing the stitches and opening the surgical incision, thereby reducing the risk of evisceration due to direct injury to the implanted fascia. For future procedures, we are considering the use of intradermal sutures in the recipients.

Observations on 7 and 11 PTD allowed us to directly assess the macroscopic state of the graft, determining the presence or absence of clinically evident rejection, and observe the development of new blood vessels over the new fascia. A limitation of this work was to be able to perform histological studies capable of quantifying the immunological processes related to TxARF to achieve a better understanding. To do so, it seems essential to do more experiments, using the model described in this work to be able to evaluate markers of tissue fibrosis, hematopoietic cells, and identify biomechanical indicators of elasticity and resistance in the graft in the short and long term. Additionally, imaging studies might be needed to visualize the wall-graft junction. As expected, of the total cells identified in the control fascias before implantation, the majority were Fibroblasts, the second most abundant cells were Myocytes; this is expected due to the remaining muscle fibers present in the tissue after dissection. The remaining cells present in the fascia were negligible in percentage. Currently, there isn’t neither an established nor accepted histological score for ARF rejection, which has been a serious problem for us in this work. A limitation is the difficulty in establishing a consistent criterion to characterize ARF with histological rejection, but it will be part of our future studies.

We monitored the macroscopic evidence of vascularization, noting histological increases in veins and arteries in the implanted fascia at both 30 and 120 PTD for both isogenic and allogeneic TxARF. Although we quantified the number of blood vessels (venous and arterial) in the grafts through Orcein staining and observed new vascular arborizations macroscopically on the implanted fascia, apparently growing from the upper and lower edges of the recipient's native abdominal wall. More studies are needed to identify the origin and arrangement of these new blood vessels. Considering that re-vascularization initially occurs through capillaries, an immunohistochemical study would be appropriate to identify CD31 and evaluate this vascularization process in TxARF. Our findings in this first proof of concept of this experimental model of TxARF in rats support the clinical observation that ARF can survive the necessary course for revascularized without experiencing ischemic events. By 30 and 120 PTD, our model further demonstrates that preserving the peritoneal layer prevents adhesions between the small intestine and the ARF, as published in the clinical setting.

## Conclusions

This work on TxARF in rats proves the feasibility of this experimental and translational model. Our research is now focused on describing the immunogenic nature of ARF and the development of progressive fibrosis as part of the natural history of the long-term engraftment of this non-vascularized graft, as well as describing the complete revascularization process. The development of this experimental model opens a perspective to better understand the success of TxARF in transplant patients but looking to further increase its clinical use and practice in general surgery.

## CRediT authorship contribution statement

**Pablo Stringa:** Writing – review & editing, Writing – original draft, Investigation, Formal analysis. **Marco Santillán Pazmiño:** Formal analysis. **Anastasios Giannou:** Writing – review & editing, Methodology. **Constanza Arriola-Benitez:** Writing – review & editing, Writing – original draft, Investigation, Formal analysis. **Martín Rumbo:** Writing – review & editing, Writing – original draft, Investigation, Formal analysis. **Virginia Gentilini:** Writing – review & editing, Writing – original draft, Investigation, Formal analysis. **Gabriel Gondolesi:** Writing – review & editing, Writing – original draft, Investigation, Formal analysis, Conceptualization. **Jeremias Elias Moreira:** Writing – review & editing, Writing – original draft, Visualization, Supervision, Software, Methodology, Investigation, Formal analysis, Conceptualization.

## Ethical clearance

The procedures were approved by the internal review board and ethics committee for the care and use of laboratory animals (CICUAL UF 2021-016 PICT 2016-3677, Ext. CICUAL UF 2019-004) and the ARRIVE 2.0 guidelines for animal studies.

## Funding statement

This research did not receive any specific grant from funding agencies in the public, commercial or not-for-profit sectors.

## Declaration of Competing Interest

None of the previously mentioned authors of the manuscript have any conflicts of interest.
